# The impact of African swine fever virus on smallholder village pig production: An outbreak investigation in Lao PDR

**DOI:** 10.1111/tbed.14193

**Published:** 2021-07-07

**Authors:** Nina Matsumoto, Jarunee Siengsanan‐Lamont, Tariq Halasa, James R. Young, Michael P. Ward, Bounlom Douangngeun, Watthana Theppangna, Syseng Khounsy, Jenny‐Ann L.M.L. Toribio, Russell D. Bush, Stuart D. Blacksell

**Affiliations:** ^1^ Sydney School of Veterinary Science The University of Sydney Camden New South Wales Australia; ^2^ Mahidol‐Oxford Tropical Medicine Research Unit Faculty of Tropical Medicine Mahidol University Bangkok Thailand; ^3^ National Animal Health Laboratory Department of Livestock and Fisheries Ministry of Agriculture and Forestry Vientiane Lao People's Democratic Republic; ^4^ Section of Animal welfare and Disease Control Department of Veterinary and Animal Sciences Faculty of Health and Medical Sciences University of Copenhagen Frederiksberg C Denmark; ^5^ Centre for Tropical Medicine & Global Health Nuffield Department of Medicine University of Oxford Oxford UK; ^6^ Lao‐Oxford‐Mahosot Hospital‐Wellcome Trust Research Unit (LOMWRU) Mahosot Hospital Vientiane Lao People's Democratic Republic

**Keywords:** African swine fever, animal health economics, Lao PDR, pig production, smallholder, village

## Abstract

African swine fever virus (ASFV) causes a deadly disease of pigs which spread through southeast Asia in 2019. We investigated one of the first outbreaks of ASFV in Lao People's Democratic Republic amongst smallholder villages of Thapangtong District, Savannakhet Province. In this study, two ASFV affected villages were compared to two unaffected villages. Evidence of ASFV‐like clinical signs appeared in pig herds as early as May 2019, with median epidemic days on 1 and 18 June in the two villages, respectively. Using participatory epidemiology mapping techniques, we found statistically significant spatial clustering in both outbreaks (*p* < 0.001). Villagers reported known risk factors for ASFV transmission – such as free‐ranging management systems and wild boar access – in all four villages. The villagers reported increased pig trader activity from Vietnam before the outbreaks; however, the survey did not determine a single outbreak source. The outbreak caused substantial household financial losses with an average of nine pigs lost to the disease, and Monte Carlo analysis estimated this to be USD 215 per household. ASFV poses a significant threat to food and financial security in smallholder communities such as Thapangtong, where 40.6% of the district's population are affected by poverty. This study shows ASFV management in the region will require increased local government resources, knowledge of informal trader activity and wild boar monitoring alongside education and support to address intra‐village risk factors such as free‐ranging, incorrect waste disposal and swill feeding.

## 1 INTRODUCTION

African Swine Fever (ASF) is a disease of domestic pigs and wild suids caused by the African Swine Fever Virus (ASFV). ASFV is a DNA virus present in all secretions, blood and tissues of affected animals (Sánchez‐Vizcaíno et al., 2019). It can survive for an extended period in the environment and in refrigerated or frozen meat products. ASFV can spread via direct and indirect contact, with domestic pig/pig, pig/tick and wild boar/environment cycles described in non‐African endemic areas (Chenais et al., 2018; Pérez‐Sánchez et al., 1994; Sánchez‐Vizcaíno et al., 2019).

ASFV is capable of distant spread across landscapes when facilitated by human transportation or management practices (Costard et al., 2009 Nurmoja et al., 2018). In naïve pigs and wild boar, clinical signs of ASFV generally follow the peracute or acute disease syndromes (Sánchez‐Vizcaíno et al., 2019). The first sign of an ASFV outbreak in a pig herd may be a small number of animals displaying clinical signs of the peracute syndrome, including depression, pyrexia and cutaneous hyperaemia, followed by death 1–4 days later (Sánchez‐Vizcaíno et al., 2019). In the acute syndrome, mortality rates can reach 100% within 7 days of clinical signs' appearance (Sánchez‐Vizcaíno et al., 2019).

**FIGURE 1:**
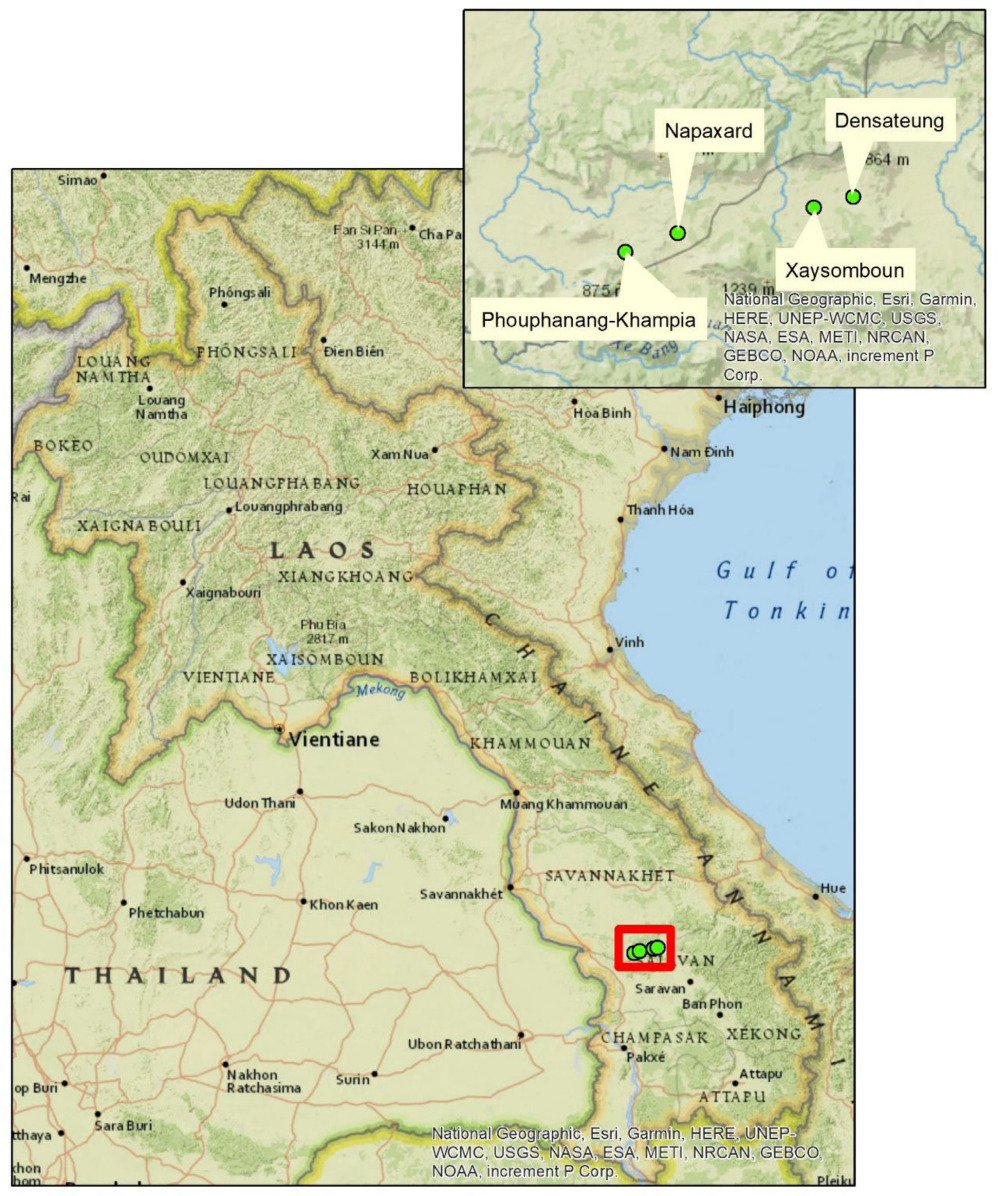
Villages studied in Thapangtong district, Savannakhet province, Lao PDR during an outbreak of African Swine Fever, 2019

Reports suggest that in 2018, contaminated swill feed carried ASFV to a Chinese pig farm, from where it spread throughout the country (Zhou et al., 2018). The disease affected all production systems, from smallholders to commercial piggeries (FAO, 2020). The disease then spread through South‐East Asia, including Vietnam, in early 2019 and was first reported in Lao PDR at the start of June 2019 (FAO, 2020). This outbreak occurred in Toomlan District, Salavane province in southern Lao PDR (FAO, 2020). A month later, in July 2019, neighbouring villages in Thapangtong district, Savannakhet province (Figure 1) first confirmed cases of ASFV (FAO, 2020).

Informal trading, low biosecurity and swill feeding – all common in Lao smallholder pig farming – increase the risk of ASFV spread (Nantima et al., 2015). Smallholder pig‐farming practices in Thapangtong are typical of lowland Lao PDR. In a previous survey of Savannakhet smallholder pig keeping practices, performed before the outbreak, median herd size was two pigs per household (Holt et al., 2019). Approximately one‐third of pigs ranged freely, and the rest were penned or tethered. Almost all pigs in the villages were either a local breed or crossbreed (94.8%) (Holt et al., 2019).

The Lao government animal disease reporting system begins at the village level: farmers report unusual outbreaks to their Village Veterinary Worker (VVW), a layperson trained in basic animal health management who reports to their District Agriculture and Forestry Office (DAFO). The DAFO communicates with their local Provincial Agriculture and Forestry Office (PAFO), which then informs the Department of Livestock and Fisheries (DLF) and the National Animal Health Laboratory (NAHL) in Vientiane. The DLF handled epidemiology and control measures, while the NAHL performed the laboratory‐based diagnosis of ASFV (Samathmanivong, personal communication, 2019). The NAHL used the TaqMan quantitative real‐time polymerase chain reaction (rt‐PCR) for confirmation of cases (King et al., 2003; Matsumoto et al., 2020).

In the 6 months from July to December 2019, ASFV spread to 17 provinces of Lao PDR, with new case numbers dramatically declining by the end of the year as the available naïve population fell (FAO, 2020). The case fatality rate averaged 85–100%, often with sudden death and/or elevated mortality as the presenting clinical sign (FAO, 2020).

Lao PDR's 2019 ASFV outbreak stretched the investigation capacity of the local veterinary services as they allocated their limited financial and human resources to national efforts in stamping out affected herds, movement controls and education programs. Globally, information on ASFV ecology and epidemiology among smallholders is sparse, particularly amongst naïve pig populations. The objective of this study was to fill this knowledge gap. As part of our activities, we allocated additional resources and time to investigate the July 2019 ASFV outbreak in Thapangtong district. In this paper we describe the ASFV outbreak, estimate related household financial loss and conduct a preliminary descriptive investigation into risk factors associated with ASFV in the Lao smallholder pig sector using data from Thapangtong district.

## 2 MATERIALS AND METHODS

### 2.1 Investigating the timeline of Lao government response

The timeline of the local government response and the process for reporting (from village to the province level) was provided by the acting head of the Savannakhet PAFO Livestock division through semi‐structured interviews conducted in English followed by a written survey.

Village Chiefs (VC) and VVW first reported abnormal pig deaths in Densateung and Phouphanang‐Khampia in late May–early June 2019 to the Thapangtong DAFO. The DAFO then reported these deaths to the Savannakhet PAFO on 25 June 2019. Together the PAFO and DAFO investigated the cases on 29 June 2019 (Samathmanivong, personal communication, 2019).

PAFO staff collected whole blood from between 1 and 4 pigs per village, using jugular venepuncture on live animals showing clinical signs. In each of the two affected villages, the PAFO team collected all samples from a single household. The samples were transported by land from the Savannakhet PAFO to the NAHL in Vientiane (Samathmanivong, personal communication, 2019). Following formal diagnosis from NAHL, PAFO and DAFO staff began control activities on 3 July. They completed stamping out measures in the two affected villages by 6 July 2019, and movement controls in the 5 km surrounding the district continued until early August 2019 (Samathmanivong, personal communication, 2019).

### 2.2 Outbreak investigation study site

This study was conducted in the Thapangtong district of Savannakhet province, which was the second location in Lao PDR to report an ASFV outbreak and is adjacent to Salavane province, where the first outbreak occurred. For this study, an ‘affected village’ was defined as a village with one or more PCR‐confirmed ASF cases. An ‘affected household’ was defined as a household that owned one or more pigs with clinical signs of ASFV in an ‘affected village’ during the high‐risk period until the end of the DLF investigation of the outbreak. Not all affected households were PCR‐confirmed.

The ‘high‐risk period’ was when the ASFV outbreak might have existed in the affected villages including the time before the first report from the Thapangtong DAFO to Savannakhet PAFO. Based on farmer interviews, clinical signs and laboratory findings, this period was estimated to be from 1 May to 2 July 2019. The period prior to viral detection was changed from the Nurmoja et al. (2018) approach used in Estonia, due to the lower resourced diagnostic setting of the study.

Of the district's three villages with confirmed cases (as at mid‐September 2019), two of similar size, Densateung and Phouphanang‐Khampia, were chosen for this study. The NAHL had confirmed the Densateung and Phouphanang‐Khampia outbreaks on 1 July 2019. Due to the high reported pig mortality rate in the affected villages, two unaffected villages, Napaxard and Xaysomboun, were selected as controls. The control villages had healthy pig populations at the time of the survey, were of similar human population size and close to the same major road as the affected villages. The study, although initially designed for traditional risk factor analysis, was changed to one that was descriptive about the impact and spread of the disease at the household level, while describing management practices at the village level. The number of surveys per village was set at 25 for simplicity of study design as a protocol needed to be created for both case and control villages.

### 2.3 Household survey

The survey had two phases: a pilot followed by a final questionnaire. Questions found to be poorly understood or in need of additional information in the pilot were adapted and included in the final questionnaire. An independent company, experienced in medical and agricultural translations, translated the questionnaire into Lao, then NAHL staff experienced in animal health extension programs back‐translated the questionnaire into English for confirmation. The questionnaire included 28 questions on how many animals they owned and their value in Lao Kip (LAK); purchasing/selling behaviour; biosecurity practices; pig management practices and pig health practices. Where literature existed about possible answers (such as housing methods and feeding), the question styles were closed. Where no literature existed, a short structured‐open question was used, such as ’How do you normally dispose of household food scraps?’ Instructions for the interviewers to guide the questioning style added clarity. The questionnaire covered the recent history of disease outbreaks in the village, including the number of animals affected and when they were affected.

Subjects were chosen from all the pig‐raising households in the selected villages. In Densateung and Phouphanang‐Khampia, almost all households were ASFV‐affected (Table 1), disease‐free pig‐owning households being extremely rare as reported by the Savannakhet PAFO. Households in the unaffected villages of Napaxard and Xaysomboun were selected as controls for comparison with the ‘affected households’ described in the previous section. Experienced animal health fieldworkers from the Savannakhet PAFO and the Thapangtong DAFO conducted the survey in late September 2019. Before the survey, they were trained in disease investigation and biosecurity practices. A few days before the planned field visit, the DAFO staff contacted the village to create a sampling frame with the VC and VVW, allowing villagers time to make themselves available on the day of surveys. The two unaffected villages were surveyed on the first day, and the two ASFV‐affected villages were surveyed on the second day. In ASFV‐affected and control villages, the VC created a sampling frame by naming 50 pig‐rearing households. The investigators randomly chose 25 representatives to interview from this list using a random number generator in Microsoft Excel (Microsoft, 2002). The VVWs and VCs also provided population‐level outbreak data and generalized spatial data. The local DAFO staff and PAFO staff conducted the surveys in Lao, with the household pig carer where available and the household head when the pig carer was not available. The questionnaires were conducted face‐to‐face in the village hall and meeting areas rather than at each household. All four villages had members of the Kattan or Bru ethnic group, some of whom did not speak Lao. These individuals worked with their VC to translate their questionnaire responses back to Lao. Most interviews took 10–15 min to complete, and each survey participant was given an educational t‐shirt as remuneration for their time.

**TABLE 1:** Herd structure by village selected for investigation in Thapangtong district, Lao PDR, showing the median number of pigs (interquartile range, total number)

	Densateung^†^	Phouphanang‐Khampia^†^	Napaxard	Xaysomboun
Piglets	6 (7.25, 176)	4 (6, 135)	1 (3, 55)	1 (1, 22)
Fatteners	0 (0, 0)	0 (0, 2)	0 (0, 5)	0 (0, 0)
Sows	1 (2, 49)	1 (1, 45)	1 (0, 25)	1 (1, 30)

^†^ ASFV‐affected village.

Boars were not included, as only one was reported in the sampled villages.

### 2.4 Participatory mapping

After the individual surveys, villagers worked with the investigators to map their village, marking their households' locations, significant landmarks and known areas of wild boar activity. This map was hand‐drawn on a large sheet of paper, and each household represented in the survey contributed to the development of the maps.

### 2.5 Data management and analysis

Data were translated into English by University‐trained animal health and laboratory staff at NAHL, stored in Microsoft Excel, collated and cleaned in Microsoft Excel and RStudio (RStudioTeam, 2018). RStudio was also used to calculate descriptive statistics on the household demography, farm details (before the outbreak), farm management and biosecurity practices (RStudioTeam, 2018). The data were then analyzed for primary epidemiologic metrics, such as epidemic curves for the survey populations and median epidemic day in RStudio using EpiR (RStudioTeam, 2018; Stevenson et al., 2019). Logistic regression was performed using the lme4 package in RStudio with the *glmer()* function and the binomial logit method (Bates et al., 2014).

### 2.6 Financial modelling

Household financial losses due to ASFV were estimated by combining the herd structure data with the estimated value of pigs, as provided by the farmers. The financial Monte Carlo simulation used the farmer‐estimated value of the pigs, multiplied by the farmer‐reported number of pigs lost. A gamma distribution (based on the survey data) was used as a prior in the *gamma.buster()* function from the EpiR package in RStudio (Stevenson et al., 2019). A Monte Carlo analysis was performed in RStudio (RStudioTeam, 2018) with 10,000 iterations to estimate the mean lost herd value with a 95% confidence interval.

### 2.7 Spatial outbreak modelling

We mapped the outbreak to investigate the spatial component of disease spread in the village. The map data were analyzed with a space–time permutation (STP) scan statistic (SaTScan; Kulldorff, 2010; Kulldorff et al., 2005). Space–time scan statistics place numerous theoretical circles of different sizes onto a map and calculate the ratio of how many disease cases are observed versus expected within each circle. The circles also extend upwards as cylinders to represent different lengths of time. The height and base are permuted across the map in all possible combinations, and all clusters are recorded (Kulldorff et al., 2005). Unlike many traditional spatial analyses, this study utilized resources from participatory epidemiology approaches. The spatial cluster analyses therefore used the hand‐drawn village maps, and the radii of the clusters used the grid (Cartesian) dimensions of the maps created.

For the SaTScan space‐time analysis, the maximum cluster size was set to 50% of the study area. The maximum period of the scanning window was set to 10 days based on the average latent period reported in the literature (Guinat et al., 2014). Monte Carlo simulation was used to determine statistical significance by running 999 replications.

## 3 RESULTS

### 3.1 Household survey

In the ASFV‐affected villages, households owned on average six piglets and two sows. None of these households owned a boar. In the control villages, households owned on average two piglets and one sow. Two of these households owned fattening pigs, and one owned a boar. All pigs in surveyed households were native breeds (Table 1).

Sampled farmers listed pig housing methods with a range of biosecurity levels, from all‐day free ranging (*n* = 38) to full‐time enclosures (*n* = 19), some of the latter being communal rather than private. Of note were the farmers who kept their pigs in enclosures near their rice paddies (*n* = 6) some distance from the village, which removed their pigs from the village ecosystem (Table 2). Reported contacts between pigs within the villages were numerous (*n* = 35 villagers confirmed contact), including with neighbours' pigs and feral pigs or wild boar. In Lao PDR, feral pigs and Eurasian wild boar are called *muu paa* (forest pig), and both closely resemble the domestic village pigs.

**TABLE 2:** Pig housing methods in Thapangtong district, Lao PDR

Housing method	ASFV‐unaffected households	ASFV‐affected households
Adults penned; piglets free to roam	5.5% (*n* = 5)	3.3% (*n* = 3)
Communal pen	5.5% (*n* = 5)	1.1% (*n* = 1)
Free range (all times)	13.2% (*n* = 12)	28.6% (*n* = 26)
Free range during day, penned at night	3.3% (*n* = 3)	0% (*n* = 0)
Multiple choices	6.6% (*n* = 6)	2.2% (*n* = 2)
Other	1.1% (*n* = 1)	3.3% (*n* = 3)
Penned (all times)	12.1% (*n* = 11)	2.2% (*n* = 2)
Rice paddies	7.7% (*n* = 7)	1.1% (*n* = 1)
Tethered near the home	1.1% (*n* = 1)	0% (*n* = 0)
Enclosure under the house	1.1% (*n* = 1)	1.1% (*n* = 1)

Only two farmers (*n* = 2) reported feeding pork or kitchen swill to their pigs. All surveyed farmers reported feeding a mixture of rice bran and the water used to prepare sticky rice as the pigs' primary diet. Water sources (other than the rice water) included household water supplies, communal wells and rivers. Of the farmers surveyed, 79 used a communal water source for their pigs and 17 used private water sources. When asked an open‐ended question about how they disposed of their kitchen rubbish, farmers gave various responses, including burying waste. However, the most common method was to burn kitchen waste. Most surveyed households butchered animals inside the house after slaughter, but 14.9% butchered animals outside the house. Many farmers gave the leftover bones to their dogs (50.7%). Another possible transmission source was using the same syringes and needles to treat multiple sick animals during the outbreak as reported by the VVWs. Several farmers attempted antibiotic therapy, and during a semi‐structured interview a VVW explained that they sometimes washed the syringe with soap and water between uses rather than disposing of the syringe.

### 3.2 Outbreak investigation

We surveyed 49 ASFV‐affected households and 50 control households. Of these 99 households, eight households surveyed in the ‘affected villages’ did not meet the definition of an ‘affected household’ (outlined in the Section 2). These households were not included in calculations relating to outbreak characteristics, outbreak losses, spatial modelling or epidemic statistics. However, these eight households were included for the purposes of describing management styles and practices. Across the ASFV‐affected households surveyed (*n* = 41), 330 pigs died with clinical signs of ASFV during the high‐risk period. No pigs died in the control villages during the same period.

### 3.3 Outbreak characteristics

During the household surveys, an obvious route of disease entry did not become apparent. Direct contact through the purchase of an infected pig seems unlikely as none of the affected farmers in this survey purchased new pigs in the high‐risk period or the 4 weeks prior. However, all (both affected and unaffected) reported Vietnamese pork traders during the risk period. Farmers were asked about their initial diagnosis, and 21% identified the cause of the deaths as a seasonal disease. However, many were unsure of the cause of the sudden increase in pig deaths (51%). The VVWs were also uncertain about what disease was causing the outbreak. An average of nine pigs died or were culled in affected households surveyed (*n* = 41). The majority of pigs died and were either buried or burned rather than culled, and only deaths recorded on or after 3 July were culled and buried by authorities.

**FIGURE 2:**
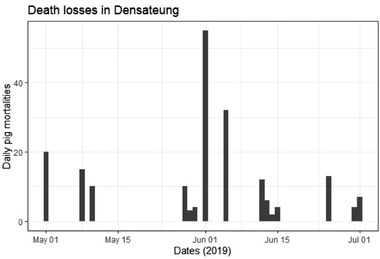
Epidemic curve for survey participants in Densateung

**FIGURE 3:**
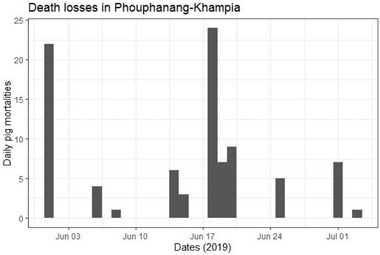
Epidemic curve for survey participants in Phouphanang‐Khampia

Of the affected animals (*n* = 330), the most common early clinical signs were depression (21.5%), fever (15%), inappetence (15%) and shivering/trembling (15%). Late clinical signs included seizures/convulsions (21.1%), shivering (11.1%) and ‘looking cold’ (6.7%). Many farmers noted death or sudden death (28.9%). The median clinical interval from onset of clinical signs to death was less than one day (IQR = 2 days), meaning that farmers observed their pigs becoming sick and dying within 24 h. The mean clinical interval was 4.4 days (SD ± 6.1). In Densateung, the median epidemic day was 1 June 2019, with an interquartile range (IQR) of 35 days. In Phouphanang‐Khampia, the median epidemic day was 18 June 2019 with an IQR of 5.5 days (Figures 2 and 3). Farmers and VVWs attempted treatments, including antibiotics (penicillin or oxytetracycline) and vitamin injections. However, farmers' records of dose, medication, frequency, age category and the route of administration were often incomplete. In the affected villages, almost all pigs died from disease before the stamping out measures commenced. Pigs that survived (*n* = 6 households) were kept in enclosures adjacent to rice paddies and were therefore not included in the stamping‐out measures.

### 3.4 Risk factor analysis

The quasi‐complete separation of ASFV outcome by the villages made the data unsuitable for logistic regression (Bates et al., 2014). When including ‘village’ as a random effect in the logistic regression model, no significant association between the odds of being an ASFV‐affected household and housing style, water source, butchering method or pig contact structure was found. The intraclass correlation attributable to the village effect was > 95% for all analyses. Smaller herds of three pigs or less approached statistical significance when taking village into account (*p* = 0.06). This is likely because smaller herds were significantly associated with the two control villages, Napaxard (*p* < 0.05) and Xaysomboun (*p* < 0.001), while the two ASFV‐affected villages had more households with larger herds.

### 3.5 Financial loss modelling

**FIGURE 4:**
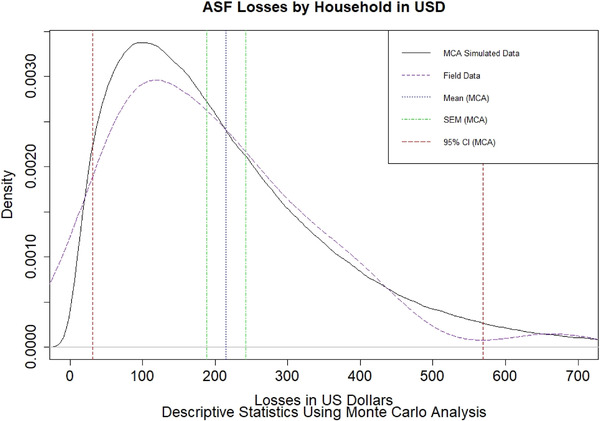
Monte Carlo analysis (MCA) of household financial losses, overlaid with original data (purple)

Modelling of the financial impact of ASFV in affected villages is presented in Figure 4 where the purple line represents the density of households' losses using the field data. The Monte Carlo simulation then drew from a gamma distribution (shape 1.85 and scale 1013712.97) created using the field data in *gamma.buster* in EpiR. After 10,000 simulations, the mean financial loss estimated in the Monte Carlo analysis was USD215.00, 95% CI (31.19, 569.30) with SEM ± USD26.85 (Figure 4).

### 3.6 Spatial outbreak modelling

**FIGURE 5:**
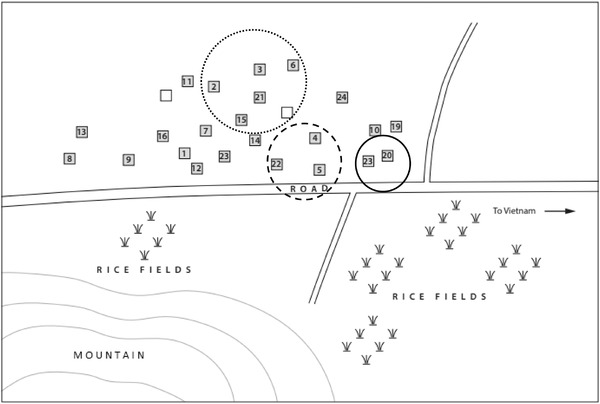
Spatio‐temporal ASFV outbreak clusters in Densateung village 2019 Solid circle – earliest dates; dashed circle – middle dates; dotted circle – later dates

**FIGURE 6:**
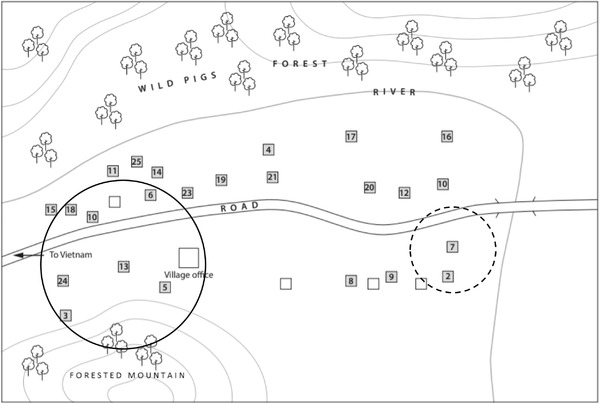
Spatio‐temporal ASFV outbreak clusters in Phouphanang‐Khampia village 2019; solid circle – earliest dates; dashed circle – later dates

Three significant clusters of more than one household and three clusters of one household (*p* < 0.001) were detected in Densateung village. The first cluster noted ASFV symptoms in the second week of May 2019 and was the earliest cluster affected in the Thapangtong region (Figure 5). Households 4, 5 and 22 accounted for 26 of the affected pigs in Densateung. This cluster was at the eastern end of the major road running through the village, which runs west‐east from Thapangtong to the Vietnam border, via Salavane province. The ensuing clusters of more than one household occurred sequentially north‐west from the first reported cluster. The outbreak in Phouphanang‐Khampia began almost a month after the outbreak in Densateung. It included two significant clusters of more than one household and four clusters of one household (*p* < 0.001) (Figure 6). The first spatial cluster involved households 13, 5, 10, 24, 3, 6 and 18 over 15–18 June, which was after the first reported household in the village.

## 4 DISCUSSION

This study describes the epidemiologic characteristics, including financial losses, associated with ASFV outbreaks in selected villages in Lao PDR. The study highlights knowledge that could be implemented to reduce the impact of ASFV and similar transboundary animal diseases on smallholders in similar resource limiting contexts. By performing this study, we also explored extant challenges and preliminary strategies to reduce the opportunity for inter‐ and intra‐village spread of ASF. These strategies will benefit policymakers and researchers beyond ASFV in the control of other high‐impact and zoonotic diseases.

The major potential pathways for introducing ASFV discussed here include traders of live pigs/pig products, iatrogenic spread and wild boar. This study did not identify any single, obvious route of ASFV entry into the villages. However, many plausible hypotheses present themselves, and all should be addressed in future disease prevention activities. The study made obvious that conditions within the villages were ideal for the spread of ASFV. A combination of inter‐ and intra‐village control measures will be required in future to prevent the spread and establishment of ASFV in smallholder communities.

A putative source for the ASFV outbreak in southern Lao PDR is the ASFV outbreak in Vietnam that began in early 2019. Both Thapangtong district and the first‐affected Toomlan district are on the same major road to Vietnam. Despite a lack of evidence that any ASFV infected live pigs were purchased from traders in the high‐risk period or the month prior, the reports of Vietnamese traders suggest increased activity from a region known to have had ASFV in that same period. Whilst the traders did not sell the villagers any pigs, the traders would have been able to contaminate the villages with ASFV contaminated pork meat products, pig wastes from trucks or even by dropping off contaminated carcasses. Previous social network analyses in the Northern Province of Xayabouri suggest that semi‐commercial piggeries interact almost exclusively with 1–2 traders (Poolkhet et al., 2019). The lack of information on trader behaviours that might cause ASFV warrants future investigation. In future studies, the social network of interactions between traders and villagers in the Southern region should be investigated to understand national and transboundary ASFV epidemiology better.

ASFV can be found in the meat, blood, urine and faeces of infected pigs and provides numerous opportunities for indirect spread (Sánchez‐Vizcaíno et al., 2019). VVWs mentioned that they had tried treating many of the symptomatic pigs, which may have led to iatrogenic spread through shared needles or insufficient disinfection between uses. Further investigation into farmer and VVW medication practices is warranted. Butchering outside after slaughter can cause significant environmental contamination during an ASFV outbreak, and several farmers in this survey participated in this practice. The movement of wild boar bones by scavenging animals has been implicated in European ASFV outbreaks. In Lao PDR, roaming dogs could be a similar indirect transmission pathway. Many farmers reported feeding leftover bones to their dogs. Despite only two farmers reporting that they fed pork waste to their pigs, opportunities for pigs to access and cannibalize ASFV‐contaminated remains resulted from household choices to bury rubbish, butcher pigs outside and spread kitchen wastes on gardens for compost. Future village education should discourage unsafe swill‐feeding practices and include safe methods of potentially infectious waste disposal and butchering.

Wild boar and feral pigs are a possible source of the ASFV outbreak described, as in European outbreaks, however current literature suggests this to be unlikely in Lao PDR (Denstedt et al., 2020). Spread of this nature would require prior evidence of ASFV circulating in wild boar populations over enough time for the disease to spread over large distances (Boklund et al., 2020; Schulz et al., 2019). The distance from the Vietnamese border to south‐central Laos is large, and wild boar facilitated spread seems unlikely given the above conditions. In this study, two farmers noted that village pigs had contact with wild ‘forest pigs’ and that the studied villages (ASFV‐affected and control) were near forests with forest pig populations. For the disease to spread from Vietnam to Thapangtong, the disease would have to have circulated in wild boar populations over a distance of approximately 168 km without affecting any other villages before Salavane and Thapangtong District. In late 2019, wild boar ASFV outbreaks were noted in the far northern province of Houaphan, meaning wild boar remain a potential future outbreak source in the wild boar‐environmental contamination pathway (Denstedt et al., 2020). However, the authors of the wild boar investigation posited that the outbreak was due to a spill over from the domestic population, and not the other way around (Denstedt et al., 2020). A recent scoping review of ASFV transmission suggests that transmission from wild boar to domestic pigs is generally unlikely. The speed of disease spread in 2019 is more suggestive of human involvement in the spread of ASFV (Barrett et al., 2020).

The nature of the outbreak made it so the data were unsuitable for risk factor analysis at the household level as initially planned. The authors initially designed the study in the assumption that not all households in the villages were going to report being an ‘affected household’, however it became apparent very quickly that the biggest risk factor for being an ‘affected household’ was being in an ‘affected village’. Because of the quasi‐complete separation of the disease outcome by village, the data was inappropriately structured for logistic regression analysis at the household level. Risk factors for ASFV transmission include free‐ranging, swill feeding and poor farm‐level biosecurity, many of which were present in both the case and the control villages. While these factors probably impact on ASFV outbreaks in Lao smallholders, it is likely that a whole village risk factor also exists. To estimate risk factors, we believe a village‐level analysis must be performed, although we recognize the difficulty of finding enough affected and unaffected villages to perform such a study. In future, a spatial mapping approach using PCR‐confirmed villages may provide opportunities to perform such an analysis in the absence of survey data.

Once ASFV entered a village, factors such as wide‐spread use of free‐ranging and generally higher pig populations allowed for the spread of the virus. Within the affected villages, a combination of direct and indirect transmission pathways facilitated the spread of disease. Sick animals could make contact both within and between herds because two‐thirds of pigs were either fully or partially free‐range. Sick pigs can spread ASFV via direct contacts, such as a sow to her piglets. Other pigs may cannibalize a sick or dead pig, and healthy pigs can eat kitchen wastes containing contaminated pork scraps. Their rooting and investigating instincts can lead pigs to uncover shallow‐buried contaminated waste or carcasses. As demonstrated in the epidemic curves (Figure 2 and Figure 3), the disease propagated through the free‐ranging and nonfree ranging pig populations once established. Of interest is the considerable difference in IQR for the epidemic days for Densateung (35 days) and Phouphanang‐Khampia (5.5 days). It appears that the smallholder village pigs in Densateung operate under a contact structure similar to those in a commercial style farm where the disease spreads slowly before causing serious fatalities. The spread of the disease amongst the pigs of Phouphanang‐Khampia more closely resembles that of a single pen of affected animals (Guinat et al., 2014). Animals with ASFV become infectious when clinical signs develop. The modal period, from clinical signs to death, in this study was one day or less. This short symptomatic period is consistent with reports of ASFV in other Asian and European outbreaks (Guinat et al., 2014; Olesen et al., 2017; Sánchez‐Vizcaíno et al., 2019; Tran et al., 2020). Future studies should estimate the *R*_0_ of ASFV transmission at the pig and household level in these villages and compare these estimates with those of commercial piggeries. The results suggest that preventing ASFV entry at the village level is likely the best strategy for protecting whole communities.

The aim of assessing the participatory data for a spatio‐temporal relationship between outbreak locations was to quantify how the disease spread through the villages beyond the calculation of an epidemic curve. The statistically significant clustering of disease outbreaks implies that the outbreak sources were not randomly distributed or a universal exposure. In particular, the sequence of localised clusters in Densateung followed a pattern moving across the village in sequentially bigger groups, reflecting the epidemic curve's propagative nature. The STP approach employed in this study, requires only case data, whereas Poisson and Bernoulli spatio‐temporal analyses require both case and population at‐risk or control data (Gatrell & Durr, 2004). For an outbreak of ASFV, the STP approach is appropriate because all pigs are affected in a village during a short period, and the population can be considered a closed cohort (Ward & Carpenter, 2000). In these low‐biosecurity, free‐ranging contexts, there are often no control households or animals. In future studies, these outputs could be adjusted by using disease parameters unique to this outbreak, estimated using approximate Bayesian computation with sequential Monte Carlo technique. Based on the strong village effect detected in the logistic regression analysis, future spatial analyses could use villages as the analytical unit to further investigate the spread of ASFV through Lao PDR.

ASFV outbreaks require prompt and thorough investigation. The epidemiologic findings suggest that ASFV was well established in the two villages before local authorities were able to act. The disease notification system used by the DLF (outlined in Section 2.1) relies on VVWs to identify and report cases to the DAFO, reporting to the PAFO for investigation. There are no standardized processes across the provinces, and funding for disease outbreak investigation is limited to the private veterinary incomes of the PAFO and DAFO staff. Weaknesses in this ‘ground‐up’ reporting approach emerged in the 2015 Vientiane FMD outbreak where numerous FMD‐affected villages that were presumed to be ‘FMD free’ by DAFO due to no reports from VVWs, yet retrospective serology determined otherwise (Miller et al., 2018). Here we note the discrepancy in the number of ASFV cases reported by the PAFO to the OIE (*n* = 80) and the number of animals with ASFV‐like clinical signs in the ‘high risk period’ (*n* = 330). The reported clinical signs, whilst typical of acute and peracute ASFV are also typical of classical swine fever, erysipelas, salmonellosis, and highly pathogenic porcine respiratory and reproductive syndrome, all of which are endemic to Lao PDR. Only five animals were sampled and definitively diagnosed as ASFV cases using PCR, highlighting difficulties in a centralized testing system. In the pilot survey, several farmers reported that pig disease was common during June and July (Lao PDR' wet season). They initially thought the deaths were due to this endemic disease syndrome. The familiar clinical signs may have also delayed reporting and action. None of the villagers included ASFV in their initial diagnoses for the pig deaths despite information materials being made available to local authorities by the OIE in early 2019.

The sampling strategy described here allowed us to speak with many household representatives in each village; however, it has its limitations. Households were selected from a list provided by the VC, who may have favoured owners that were more educated, more receptive to government communications, owned more pigs or had positive management traits, leading to selection bias. Herd size skewed right across all villages, with most households owning small herds of three to four pigs and a few exceptional individuals owning larger herds. The ASFV‐affected villages had more households with large herds than the control villages (Table 1). The relationship between village and herd size suggests that disease entry into the village could be related to increased economic activity. This contrasts with evidence from Uganda, where ASFV is endemic and socioeconomic impact surveys in 2014–2015 found that smallholder households with larger herds were significantly associated with larger economic outputs and lower incidences of ASFV (Chenais et al., 2017). There is a possibility that whilst having a larger herd is protective to the household, it is a risk factor at the village level to have numerous large herds. This observation bears further investigation in future village level analyses.

Discussions on the impact of the 2019 ASFV outbreak on global markets have focused on pork prices, demand for alternative sources of protein and demand for intensive livestock feed products such as soya beans (Croz et al., 2020). Here we have estimated the cost to the smallholders and their local communities, a neglected aspect of the epidemic. Of Laotians affected by poverty in 2018–2019, 20–30% live in the neighbouring provinces of Savannakhet and Salavane (World Bank, 2020). Thapangtong district is located at the border between the two provinces, and 40.6% of its population were living in poverty in 2015 (Coulombe et al., 2016). In neighbouring Toomlan district, where the outbreak began, 73.1% are affected by poverty (Coulombe et al., 2016). The modelled losses from ASFV of USD 215 per household are a substantial portion of annual income for smallholders in the region, who are already at risk of food insecurity due to economic shocks and environmental disasters. The wide confidence intervals (USD 31.19, 569.30) with smaller SEM (± USD 26.85) suggest that the data set was not limited by size, but rather that there is substantial heterogeneity in the economic impacts and herd structures of smallholder farmers. However, this estimate is based solely on the sale value of pigs that died. The method calculated the minimum possible loss as it was restricted to the value of the pigs alone. It did not consider lost treatment costs, time, future value or social costs. A more extensive study could estimate gross margins by calculating the production costs, based on more extensive interviews with farmers and collecting data on inputs, outputs and uses for dead pigs. Other studies have reported that dead or diseased pigs may not have immediately lost their monetary value – as farmers may have sold the meat or kept it for household consumption (Chenais et al., 2017) – but this behaviour was not reported in our survey. During the survey, questions about medication costs, vaccination and feeding received few responses, suggesting a very low‐output/low‐input system. This might explain why farmers continue to purchase and raise smallholder pigs despite risks of high‐impact transboundary animal diseases.

The findings of this study should be utilized in future decisions about the management of ASFV in the region. Trader, VVW and villager behaviour must be managed and control measures put into place for contact between village pigs and wild boar. The resources available to local government authorities must be assessed for them to act promptly in cases of emergency disease outbreaks. When designing control and education strategies, local farming practices, as well as the disease ecology must be considered together in order to develop effective materials to aid in the prevention and management of ASFV outbreaks into the future.

## CONFLICT OF INTEREST

The authors declare that there were no conflicts of interest in the development of this work.

## ETHICS STATEMENT

The authors confirm that the ethical policies of the journal, as noted on the journal's author guidelines page, have been adhered to. Ethics approval for the surveys was obtained from the University of Sydney Human Ethics committee, approval number [2019/725].

## Data Availability

The data that support the findings of this study are available on request from the corresponding author. The data are not publicly available due to privacy or ethical restrictions.
